# System biology prediction model based on clinical data: highly accurate outcome prediction in patients with acute-on-chronic liver failure

**DOI:** 10.1186/cc10996

**Published:** 2012-03-20

**Authors:** MJ McPhail, DL Shawcross, RD Abeles, T Chang, GL Lee, MA Abdulla, C Willars, E Sizer, G Auzinger, W Bernal, JA Wendon

**Affiliations:** 1 King's College Hospital, London, UK

## Introduction

Present outcome prediction tools for patients with acute-on-chronic liver failure during critical illness are only of moderate accuracy. Regression methods on latent variables (usually applied to top-down system biology applications with spectroscopic data) may offer significant advantages over logistic regression techniques as multiple cross-correlations are acceptable in this form of modelling.

## Methods

Between 1 January 2000 and 31 December 2010 all patients admitted to the Liver Intensive Therapy Unit (LITU) at King's College Hospital had daily prospective collection of demographic, biochemistry and bedside physiology. Logistic regression modelling (LRM) and partial least-squares discriminant analysis (PLSDA), Model for End-stage Liver Disease (MELD) and APACHE II scores were compared using receiver operating characteristic (ROC) curve analysis.

## Results

A total of 986 patients (median age 52 (range 16 to 90) years; 603 (62%) male) with cirrhosis and emergency LITU admission were identified. The median APACHE II score was 21 (5 to 50) and the median MELD score 23 (3 to 50). Overall LITU survival was 63% and survival to hospital discharge 51%. Predictive accuracy at day 3 was improved in all models over admission values. The AUROC for LITU survival for MELD and APACHE scores on day 3 was 0.78 (95% CI 0.75 to 0.82, sensitivity 72%, specificity 75%) and 0.83 (0.78 to 0.83, sensitivity 83%, specificity 63%) respectively. A LRM utilising nine variables had an AUROC of 0.85 (95% CI 0.82 to 0.87, sensitivity 72%, specificity 83%). Two-component PLSDA identified 30 variables with independent prognostic significance. Performance in outcome prediction was improved over logistic regression at day 3 - sensitivity 86%, specificity 81%, AUROC 0.91 (0.89 to 0.93, *P *< 0.001 for all comparisons) in a model incorporating 30 variables. Cross-validation and permutation analysis confirmed the internal validity of this method.

## Conclusion

This application of latent variable regression modelling techniques to intensive care datasets demonstrates high accuracy of prediction. Liver-specific outcome schema based on logistic regression may not fully describe the complex cross-correlating interactions that PLS techniques are designed to incorporate. Further validation in other centres and disease groups is warranted.

